# Neopterin in Diagnosis and Monitoring of Infectious Diseases

**DOI:** 10.1155/2013/196432

**Published:** 2013-12-08

**Authors:** Michael Eisenhut

**Affiliations:** Paediatric Department, Luton and Dunstable University Hospital NHS Foundation Trust, Lewsey Road, Luton LU40DZ, UK

## Abstract

Neopterin is produced by activated monocytes, macrophages, and dendritic cells upon stimulation by interferon gamma produced by T-lymphocytes. Quantification of neopterin in body fluids has been achieved by standard high-performance liquid chromatography, radioimmunoassays, and enzyme-linked immunosorbent assays. Neopterin levels predict HIV-related mortality more efficiently than clinical manifestations. Successful highly active antiretroviral therapy is associated with a decrease in neopterin levels. Elevated neopterin levels were associated with hepatitis by hepatitis A, B, and C viruses. Serum neopterin levels were found to be a predictor of response to treatment of chronic HCV infection with pegylated interferon combined with ribavirin. Neopterin levels of patients with pulmonary tuberculosis were found to be higher in patients with more extensive radiological changes. Elimination of blood donors with elevated neopterin levels to reduce risk of transmission of infections with known and unknown viral pathogens has been undertaken. Neopterin measurement is hereby more cost effective but less sensitive than screening using polymerase chain reaction based assays. In conclusion neopterin is a nonspecific marker of activated T-helper cell 1 dominated immune response. It may be a useful marker for monitoring of infectious disease activity during treatment and for more accurate estimation of extent of disease and prognosis.

## 1. Introduction

Neopterin was first isolated from larvae of bees, in worker bees and in royal jelly in 1963, and subsequently from human urine by Sakurai and Goto in 1967 [1].

**Figure 1:**
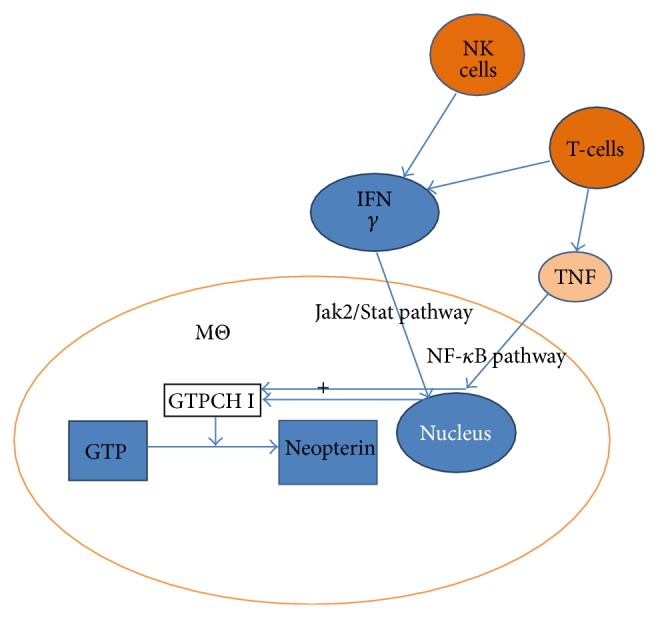
Pathways for induction of neopterin production (MΘ: macrophage, Jak: Janus kinase, Stat: Signal Transducer and Activator of Transcription, and GTPCH: guanosine triphosphate cyclohydrolase).

Neopterin or 2-amino-4-hydroxy-6-(D-erythro-1′,2′,3′-trihydroxypropyl)-pteridine is produced from guanosine triphosphate via guanosine triphosphate cyclohydrolase I (GTPCH I) by activated monocytes, macrophages, dendritic cells, and endothelial cells and to a lesser extent in renal epithelial cells, fibroblasts, and vascular smooth muscle cells upon stimulation mainly by interferon gamma and to a lesser extent by interferon alpha and beta with its release being enhanced by tumor necrosis factor [2, 3]. GTPCH I mRNA expression is synergistically and independently induced by interferon gamma through the Jak2/Stat pathway of nuclear transcription regulation and through TNF by the NF-kappaB pathway (see Figure 1) [4]. Release in response to cytokines released by T-lymphocytes and natural killer cells make neopterin an indicator of activation of cell mediated immunity including release by infections associated with activation of T-lymphocytes and natural killer cells, malignancies, autoimmune diseases, rejection of transplanted organs, and atherosclerosis.

At its first isolation in the 1960s neopterin was detected in the pupae of bees by anion exchange chromatography followed by paper chromatography [1]. In the seventies gas chromatographic-massfragmentographic methods were described allowing measurement in urine. Subsequently detection and quantification of neopterin succeeded in serum, urine, and other body fluids using standard high pressure and by reverse-phase high-performance liquid chromatography with fluorescence detection. Later simpler radioimmunoassays and more recently enzyme-linked immunosorbent assays have been developed which are suitable for large numbers of samples [1]. Semiquantitative measurement with a dipstick system using polyclonal antineopterin antibodies has been validated and may be suitable for bedside testing and in the setting of developing countries [5].

## 2. Neopterin Release in Viral Infections

### 2.1. Relationship of Neopterin Levels to Time Course of Viral Infections

During acute viral infections increased neopterin levels have been observed, which correlate with the activity of disease. This was first described in 1979 [6] and subsequently neopterin elevations were noted in infections with hepatitis viruses, Epstein-Barr, Cytomegalo, measles, mumps, varicella zoster, rubella, and influenza viruses [1, 7–11]. Elevated neopterin levels in body fluids were found at the end of the incubation period before the onset of clinical symptoms. The highest neopterin levels occur just before specific antibodies against the virus become detectable, which is about two to four weeks after onset of increased neopterin production. In acute varicella zoster virus infection peak neopterin levels were observed at the end of the appearance of the rash and in measles virus infection one to three days after appearance of the rash [12, 13]. Immunisation with live viruses, for example, measles, mumps and rubella and virus vaccine, resulted in a significant increase of neopterin independent of presence of any symptoms. In measles vaccination neopterin levels were observed to rise at a median of 5 days after vaccination about 7 days before the appearance of antibodies [13]. These investigations point to a future application of measurements neopterin as a correlate of a successful vaccination. Neopterin should be investigated as a marker to evaluate protective efficacy of vaccines stimulating cell mediated immunity against mycobacterial, parasitic, or viral diseases. The magnitude of the elicited neopterin levels could be put into relationship to incidence of the disease immunised against the population of immunised children.

Serum neopterin levels were also found to be significantly elevated in symptomatic dengue virus infections with levels higher than in measles and influenza virus disease [14]. Levels correlated with duration of fever and severity of disease [14, 15].

Investigations into the physiological functions of neopterin in viral infections revealed that it is able to delay the development of the cytopathic effect of coxsackie B5 virus in Hep-2 cells [16]. A proposed mechanism is the stimulation of inducible nitric oxide synthase expression leading to an increase in nitric oxide production. Other mechanisms include the induction of the translocation of the nuclear factor-kappa B to the nucleus.

### 2.2. Neopterin as Diagnostic and Prognostic Marker in HIV Infection

Testing of 328 samples of 29 HIV infected individuals found that 44/68 (64.7%) of samples, which were HIV-1 RNA and p24 antigen positive had elevated neopterin levels (>10 nmol/L). 6/216 (2.8%) samples, which were both HIV-1 and p24 antigen negative had elevated neopterin levels [17]. Neopterin levels were also found to be significantly elevated in HIV-2 infection compared to controls [18].

Studies investigated markers of immune activation for their usefulness as prognostic markers in HIV infection and showed an increase of neopterin levels in people with HIV infection compared to patients without HIV infection [19–22]. Neopterin levels hereby were found to increase early in the course of HIV infection preceeding CD4+-T-cell decline and clinical manifestations of AIDS [23, 24]. Plasma neopterin levels were found to correlate with plasma HIV viral load [25].

Neopterin levels were found to predict HIV related mortality [26, 27]. A retrospective study compared *β*_2_ microglobulin, immunoglobulin A, G, and M, adenosine deaminase, and neopterin levels above normal range as predictors of clinical or immunological deterioration in 256 patients with HIV infection. Changes in *β*_2_ microglobulin levels showed the greatest sensitivity to detect worsening (43%) with neopterin slightly less sensitive (41.9%) followed by immunoglobulin levels (26.8–35.2%) and adenosine deaminase levels with 21.8% having the lowest sensitivity [28].

### 2.3. Neopterin as Surrogate Marker for Viral Load to Monitor Response to Antiretroviral Treatment

In a land mark study the effects of dual reverse transcriptase inhibitor (RT) therapy and highly active antiretroviral therapy (HAART) on neopterin levels in patients with HIV infection were compared to HIV uninfected controls, HIV infected patients not on treatment, and patients who had stopped treatment [22]. RT inhibitor treatment decreased circulating levels of neopterin (mean of 15.6 for treated versus a mean of 22.3 ng/mL for untreated HIV patients, *P* < 0.04). HAART decreased neopterin levels significantly further. This confirmed results of a previous study on the effects of HAART on neopterin levels [29]. Neopterin levels in patients who discontinued HAART became similar to untreated HIV patients.

Neopterin may be a particularly useful surrogate marker for monitoring of control of HIV replication in settings in developing countries where HIV RNA viral load measurement is not available and may be a cheaper alternative particularly if semiquantitative dip stick tests are used for urine samples [5]. Longitudinal serial measurements in the same individual could overcome difficulties with interpretation in settings where chronic parasitic (malaria) or bacterial (tuberculosis) infections may elevate the baseline neopterin level and could allow monitoring of response to antiretroviral treatment in the absence of resistance testing and provide means to monitor compliance in the outpatient setting (see Table 1 for list of diseases in which neopterin levels have been used to monitor treatment response).

### 2.4. Viral Hepatitis

The first study investigating the role of neopterin in specific forms of viral hepatitis tested urinary levels in patients with hepatitis A, hepatitis B, and non-A, non-B hepatitis virus infection [9]. The authors noted that in 51 patients with acute viral hepatitis 49 patients had elevated urinary neopterin levels with the highest levels found in patients with acute hepatitis A. While all patients with active hepatitis B had elevated neopterin levels, 49/62 HbsAg carriers (77%) had normal urinary neopterin levels. The authors noted that neopterin levels were not a reflection of hepatocellular damage as 3 patients with alcoholic hepatitis had normal urinary neopterin levels.

In order to address the question whether neopterin is a useful marker for early detection of viral infection in donated blood products before seroconversion, one study investigated neopterin levels in anti-HCV-negative specimens, which were HCV RNA and HCV core antigen positive. The investigators found that 8/217 (3.7%) had elevated neopterin levels (>10 nmol/L). 4/115 (3.5%) specimens positive for HBV DNA had elevated neopterin levels [17]. In 106 patients with thalassemia major receiving multiple blood transfusion significantly more patients with histologically proven chronic hepatitis (19/21 were anti HCV antibody positive) had elevated blood neopterin levels compared to patients with siderosis of the liver [45]. Alanine aminotransferase levels in HCV infected persons correlated significantly with neopterin levels. Serum neopterin levels were found to be a useful predictor of response to treatment of chronic HCV infection with pegylated interferon combined with ribavirin. Neopterin concentrations were evaluated in 260 HCV patients treated by pegylated interferon combined with ribavirin. Mean and median pretreatment neopterin concentrations were lower in patients with sustained virological response than in nonresponders. The rate of response was twofold higher among patients with pretreatment neopterin levels *<*16 nmol/L than in patients with neopterin levels *≥*16 nmol/L, even after controlling for HCV genotype status [38].

A recent study investigated specifically whether serum neopterin levels can discriminate between patients with replicative (*n* = 30) and nonreplicative (*n* = 25) HBV carriage [46]. Replicative HBV carriage was defined as HBV DNA >5 pg/mL by hybrid capture system. Neopterin levels had a mean of 14.5 nmol/L in replicative versus 8.8 nmol/L in nonreplicative HBV carriers (*P* < 0.05). This result was not reproducible in another study, which found that in patients with replicative HBV infection (*n* = 30) mean serum neopterin level was 24.73 nmol/L and in nonreplicative HBV (*n* = 30) 14.8 nmol/L a difference, which was not statistically significant [47]. This may have been due to large standard deviations and small numbers in groups. A more recent investigation found that in chronic hepatitis the mean ± SD serum neopterin levels were 14.2 ± 5.6 nmol/L, 20.3 ± 7.9 nmol/L in patients with liver cirrhosis and 5.2 ± 1.4 nmol/L in the control group. Serum neopterin levels were significantly higher in patients with chronic hepatitis (*P* = 0.005) and cirrhosis patients (*P* = 0.008) than in control subjects. Cirrhotic patients had significantly higher serum neopterin levels than patients with chronic hepatitis (*P* = 0.004). There was a positive correlation between serum neopterin levels and alanine aminotransferase levels in patients with chronic hepatitis (*r* = 0.41, *P* = 0.004) and cirrhotic patients (*r* = 0.39, *P* = 0.005). Positive correlations were detected between serum neopterin levels and inflammatory score in patients with chronic hepatitis (*r* = 0.51, *P* = 0.003) and cirrhotic patients (*r* = 0.49, *P* = 0.001) [48].

### 2.5. Viral Respiratory Tract Infections

Neopterin has been investigated as a marker to distinguish viral from bacterial lower respiratory tract infections. The investigators found that serum neopterin levels were elevated (>10 nmol/L) in 96% of patients with viral LRTI. The median serum neopterin concentration was almost 2-fold higher in the viral LRTI group than bacterial LRTI patients (30.5 versus 18.7 nmol/L) and 5-fold higher than those in healthy controls. The specificity for correct identification of viral LRTI was 69.5% for a cut-off of >15 nmol/L [49].

Serial monitoring of serum neopterin levels in patients with severe acute respiratory syndrome (SARS) associated virus revealed that all (*n* = 129) investigated patients had elevated neopterin levels by day 9 [50]. Duration of pyrexia in SARS patients correlated positively with neopterin levels. Patients on steroid therapy had significantly lower neopterin levels. Measurement of neopterin in isolation and in relationship to other inflammatory markers like procalcitonin and C-reactive protein were investigated for discriminatory power between viral and bacterial lower respiratory tract infections. Investigators used the CRP/neopterin ratio (C/N ratio) to discriminate viral and bacterial etiology of respiratory tract infections. In a study conducted in Hong Kong sera obtained on the day of hospitalization for LRTI from 139 patients with confirmed bacterial etiology and 128 patients with viral etiology were examined. A further 146 sera from healthy Chinese subjects with no infection were included as controls. The area under the receiver operating characteristic (ROC) curve (area under curve [AUC]) for distinguishing bacterial from viral infections was 0.838 for CRP and 0.770 for PCT. The AUC for distinguishing viral from bacterial infections was 0.832 for neopterin. When the markers were used in combination, AUC of ROC of the C/N ratio was 0.857, whereas (CRP × PCT)/neopterin was 0.856 [49]. In a subsequently reported study the median of the C/N ratio was 10 times higher in patients with bacterial aetiology than with viral aetiology (12.5 versus 1.2 mg/nmol; *P* < 0.0001) and 42 times higher than those in healthy subjects (12.5 versus 0.3 mg/nmol; *P* < 0.0001). The area under the receiver operator characteristic curve for the C/N ratio was 0.840. A cut-off value of  “C/N ratio >3” for ruling in/out bacterial/viral infection yielded optimal sensitivity and specificity of 79.5% and 81.5%, respectively [51].

### 2.6. Viral Central Nervous System Infections

Early studies showed elevated neopterin levels in cerebrospinal fluid (CSF) of patients with aseptic meningitis and herpes simplex and measles virus encephalomyelitis [52–54]. CSF levels of neopterin seem to reflect intrathecal production by microglia as pterins have a low permeability across the blood brain barrier with a serum-to-CSF distribution at a quotient of 1/40 [55]. It has recently been established that normal CSF neopterin is brain-derived. The interindividual variation of CSF neopterin in healthy adults was found not to depend on serum neopterin concentration variation (coefficient of variation, CV-CSF = 9.7% < CV-serum = 24.5%). Additionally individual normal CSF neopterin concentrations were found to be invariant to the variation of the albumin quotient, QAlb; that is, CSF neopterin does not derive from leptomeninges [56]. Patients with viral meningitis had elevated CSF neopterin levels compared to healthy controls but normal serum levels [54]. CSF neopterin levels correlated hereby with CSF monocytic cell count. Patients with various forms of encephalitis including those caused by herpes simplex virus, varicella zoster virus, and tick borne encephalitis virus had significantly elevated CSF neopterin levels compared to controls and higher levels than in patients with viral meningitis without overlap of levels in the two conditions. In HIV infection there was a clear relationship between the severity of AIDS-related dementia and CSF neopterin levels [12, 30–32]. Higher CSF HIV viral loads were associated with higher CSF neopterin levels [33].

After commencement of combination antiretroviral therapy (ART), CSF neopterin decreased markedly but remained slightly above normal levels in a substantial number of patients despite several years of receiving ART [32, 34–36]. Even patients with systemic virological failure exhibit a substantial reduction of CSF neopterin concentrations, though above that of virologically suppressed patients [37]. In patients on combination ART, the lowest CSF neopterin levels have been found in patients with the lowest CSF viral loads (<2.5 copies/mL) [32]. No significant difference in CSF neopterin concentrations was found between those treated with protease inhibitor- and nonnucleoside reverse transcriptase based regimens in combination with 2 nucleoside analogues [57]. This would support the idea that viral replication within or close to the CSF, at least to some extent, is partly driving the inflammatory response. It has also been suggested that an inflammatory response, once triggered, may lead to a self-sustaining state of cellular activation as has been seen in patients with herpes simplex virus type-1 encephalitis [58]. Findings in this study are consistent with these reports. HIV RNA levels measured in CSF or plasma were not significantly associated with CSF neopterin trajectories. In addition, all study participants had experienced virologic control to the limit of standard detection as a result of their treatment and CSF neopterin levels were the only factor strongly associated with subsequent decay rates and the ultimate set-point levels [32].

## 3. Neopterin Levels in Bacterial Infections

Patients with bacterial infections with species other than mycobacteria showed significantly lower urinary neopterin levels compared to patients with viral infections in one study [59] but no statistically significant difference in a more recent study [60]. Within the group of bacterial infections it was shown that patients with symptoms for at least 5 days had significantly higher neopterin concentrations than patients with acute illness. This applied particularly to bacterial pneumonia. Patients with urinary tract infections were found to have similar levels to patients with viral infections with data on urinary neopterin concentrations but not serum concentrations. Thus it remains unclear whether local production of neopterin takes place in urinary tract infections and serum neopterin would stay low. There was no significant difference in neopterin levels between patients with febrile neutropenia and underlying haematological and oncological conditions and gram-negative versus gram-positive infections [61]. In patients on an intensive care unit with sepsis and septic shock urinary neopterin/creatinine ratios were found to be significantly higher compared to patients with other forms of systemic inflammatory responses syndromes [62] and serum neopterin levels were higher in nonsurvivors compared to survivors of sepsis and multiorgan failure scores correlated with neopterin levels [63–66]. In this context it was however noted that neopterin levels correlated negatively with reduced renal function reflecting renal failure causing a reduced excretion of neopterin. Future studies could correct for reduced excretion due to reduced renal function by calculation of the serum neopterin/creatinine ratio.

Investigations on critically ill patients on intensive care units evaluated neopterin levels as tool to discriminate patients with systemic inflammatory response syndrome with and without infectious etiology. Neopterin levels were found to have a specificity of 78% for discriminating infectious and noninfectious etiology of critical illness [66].

Bacterial meningitis was associated with both elevated serum and CSF neopterin levels compared to controls [55]. In Lyme neuroborreliosis—a late complication of infection by the tick-born spirochete *Borrelia burgdorferi*—high neopterin concentrations were found in CSF of patients, whereas serum neopterin levels were not markedly increased, confirming intrathecal neopterin production [67]. Infection with *Treponema pallidum* subsp*. pallidum* (syphilis) was not associated with elevated neopterin levels [18]. In melioidosis by *Pseudomonas pseudomallei* neopterin concentrations were found to be significantly higher than controls [12].

In brucellosis neopterin levels were with a mean 52.5 mmol/mL significantly higher than healthy controls and patients with tuberculosis [68]. In leprosy caused by *Mycobacterium leprae* 75% of patients with tuberculoid and lepromatous leprosy presented with elevated urinary neopterin excretion [69].

### 3.1. Neopterin Levels in the Course of *Mycobacterium tuberculosis* Infection

On the basis of in vitro and in vivo data showing that macrophages release neopterin in response to stimulation by T-lymphocytes [70, 71] Fuchs et al. [39] investigated urinary neopterin levels by HPLC in 55 patients with culture confirmed pulmonary tuberculosis and compared them with 417 normal controls. 83% of patients had levels above the upper tolerance limit (containing with 95% probability 97.5% of healthy controls). Neopterin levels were higher than age and gender matched controls for every extent of pulmonary disease and correlated with its extent. The correlation with extent of pulmonary tuberculosis was also demonstrated for serum levels and levels found in bronchioalveolar lavage fluid [72]. Subsequent studies showed higher levels of neopterin in serum and pleural effusions of patients with pulmonary tuberculosis compared to controls [73, 74]. Peripheral blood mononuclear cells (PBMNC) from tuberculosis patients showed a significantly higher spontaneous production of neopterin. Stimulation with phytohaemagglutinin or purified protein derivative did not yield higher neopterin production in PBMNC of patients with tuberculosis showing that it is not the production capacity for neopterin which is different [74].

Elevated serum neopterin levels were also found in HIV infected patients with tuberculosis and decreased significantly on antituberculous treatment [40]. A relapse of tuberculosis was in 2 cases characterized by increase in neopterin levels.

Further studies compared neopterin levels in urine, serum, and bronchoalveolar lavage fluid and found that they correlate significantly in patients with tuberculosis [72, 75]. The elevation of neopterin in patients with tuberculosis was more pronounced in urine than in serum or bronchoalveolar lavage [75].

### 3.2. Differentiating Pulmonary Tuberculosis from Other Lung Diseases

Patients with pulmonary tuberculosis had significantly higher urinary neopterin levels compared to patients with lung cancer or pneumonia with more than twice the concentration reported in adults [74]. Pleural fluid neopterin levels were investigated for its ability to differentiate between tuberculous and malignant pleural effusion and were found to be significantly higher in patients with pleural tuberculosis but performance characteristics including receiver-operating characteristics curve analysis was inferior to adenosine deaminase [76].

### 3.3. Monitoring Response to Antituberculous Treatment

Waiting for the results of susceptibility tests to select an effective antituberculosis drug regimen often causes a delay in effective treatment which can be disastrous, especially in children. Because the X-ray changes tend to resolve very slowly and may get even worse after starting therapy because of a paradoxical reaction due to immune-reconstitution even in otherwise immune-competent patients, other than clinical status there is no reliable parameter to reflect success or failure of the drug regimen [77].

Urinary neopterin levels declined on twice weekly measurements in all monitored patients with pulmonary tuberculosis on treatment and fell to below tolerance limits within 10 weeks of treatment in 6/10 patients [39]. Measurement of serum neopterin levels in patients on treatment for microbiologically confirmed pulmonary tuberculosis confirmed this observation and showed a significant decline of levels to near normal levels within 6 months of treatment [41]. In the context of emerging multiple drug resistance and difficulties in monitoring compliance and drug absorption neopterin needs to be explored as a tool for monitoring of success in treatment of *Mycobacterium tuberculosis* infection. It may also help to distinguish active from latent disease. People with HIV-m. tuberculosis coinfection with active tuberculosis responded with a reduction of plasma neopterin to antituberculotic treatment but neopterin levels remained above the baseline levels of HIV negative tuberculosis patients and levels were higher in patients with lower CD4 count [78].

## 4. Significance of Neopterin in Parasitic Infections

The first study of neopterin levels in parasitic infections included measurements of urinary neopterin by HPLC in patients with *Plasmodium falciparum* and *vivax* infections including patients with low grade parasitemia [7]. All patients had elevated urinary neopterin levels compared to uninfected controls to a level of 664 to 5189 micromol neopterin/mol creatinine. Levels in patients treated with quinine sulphate and levels in untreated patients were not significantly different.

A subsequent detailed interventional study provided data on urinary neopterin levels in volunteers experimentally infected with *Plasmodium falciparum* [79]. Serial monitoring revealed that urinary neopterin levels were not elevated until peripheral blood parasite densities had increased through 3 to 4 cycles of intraerythrocytic schizogony. A sharp rise in urinary neopterin was detectable at the beginning of day 14 after infection. There was an increase one day after onset of fever. In one patient a urinary neopterin increase was noted without the occurrence of fever. Neopterin production in falciparum malaria seems to be a direct effect of plasmodial antigens on monocytes/macrophages. In vitro studies showed that the monocytic cell line U937 could be stimulated to produce neopterin with lysates of *Plasmodium falciparum* parasitized human erythrocytes and recombinant *P. falciparum *proteins [80]. At a cut-off point of 10.0 ng/mL, neopterin had a positive and negative predictive value of 0.38 and 0.98 for detection of severe falciparum malaria [81]. Chloroquine treatment was followed by a reduction of urinary neopterin levels. When clinical disease resolved within 3–7 days of treatment, neopterin levels normalized rapidly [42]. Neopterin levels in nonimmune patients and young children were higher than were those of semiimmune individuals.

CSF (CSF) neopterin levels were investigated for ability to discriminate between different stages of cerebral *Trypanosoma (T.) brucei (b.) gambiense* infection.

In an investigation of 512 *T. b. gambiense* patients originating from Angola, Chad, and the Democratic Republic of the Congo CSF IgM and neopterin were the best in discriminating between the two stages (S1 and S2) of disease with 86.4% and 84.1% specificity, respectively, at 100% sensitivity. When a validation cohort (412 patients) was tested, neopterin (14.3 nmol/L) correctly classified 88% of S1 and S2 patients, confirming its high staging power [82]. Serum neopterin was also assessed as a disease marker in human *Schistosoma mansoni *infection and levels were found to reflect the extent of hepatic involvement with higher levels found in patients with hepatomegaly. Treatment with praziquantel led to a normalisation of serum neopterin levels as a result of a reduction of egg induced immunopathology [43, 44, 83].

## 5. Application of Measurement of Neopterin in Screening of Blood Products for Infections

The detection of new blood borne viruses including HIV and non-A non-B hepatitis viruses led to investigations into new ways of excluding transmission of blood borne viruses by transfusion of blood products. The government of Tirol in Austria introduced routine measurement of neopterin levels in all donated blood in 1986.

A cut-off of 10 nmol/L was used and led to the exclusion of 1.6% of donors (total number of donors = 76587). The most common cause of elevated levels was in 123 (67%) of cases a viral respiratory tract infection. 6 donors with elevated neopterin had an acute toxoplasmosis and 4 had HIV infection or non-A-non B-hepatitis [84]. In another study 5.26% of 1767 donations with increased neopterin levels were positive for CMV IgM indicating acute infection. 0.3% of patients with low neopterin levels had CMV IgM. Seroconversion was detected in 10 patients with initially elevated neopterin levels on a second serum sample indicating that neopterin may precede the appearance of CMV antibodies by 2–4 weeks [85]. A further study of Austrian blood donors showed that neopterin levels were significantly higher in early compared to late infection or carrier state. All early infections (seroconversions) had elevated neopterin levels while only 17% of late and carrier states [86]. A recent study found that using a neopterin ELISA 61% of CMV DNA-positive samples had elevated neopterin levels [87].

With regard to other viruses 5.5% of donors with above normal neopterin had Epstein-Barr virus IgM generating an almost threefold greater chance of acute EBV infection in donors with increased neopterin (odds ratio: 2.85 (95% confidence interval, 1.5–5.6). With regard to parvovirus B19 infection 73/1060 (6.9%) donors were found to be seropositive for parvovirus B19 IgM [88]. A later study by the same group found no HPV DNA positive results amongst 1200 patients with normal neopterin levels [89].

An investigation of the association of neopterin levels with chronic hepatitis C virus infection revealed that significantly more patients with elevated neopterin levels and HCV antibodies were HCV PCR positive for HCV RNA (odds ratio: 3.76 *P* = 0.002) [90].

## 6. Conclusions

Neopterin is a nonspecific marker of activated cell mediated immunity involving release of interferon gamma. Neopterin may be a useful marker for more accurate estimation of extent of disease and hence prognosis. Knowledge of all potential causes of its elevation can overcome problems with reduced specificity in a patient known to have a specific infectious disease. Longitudinal serial measurements in the same individual could overcome difficulties with interpretation in settings where chronic parasitic (malaria) or bacterial (tuberculosis) infections may elevate the baseline neopterin level and could allow monitoring of response to antiretroviral, antituberculous, and antiparasitic treatment in the absence of resistance testing and provide means to monitor compliance in the outpatient setting (see Table 1). This is particularly important in the current context of emerging multiple drug resistance of HIV and mycobacterium tuberculosis.

Neopterin for which high quality ELISA systems to measure urine and blood levels are commercially available is an underused marker in clinical practice and is suitable for introduction into the routine clinical laboratory practice.

## Figures and Tables

**Table 1:** Applications of neopterin measurements for monitoring of treatment response in infectious diseases.

Infection	Treatment	Type of sample	Change in neopterin levels observed	Reference
Human immunodeficiency virus	Reverse transcriptase inhibitors and highly active antiretroviral treatment	Blood, cerebrospinal fluid	Mean of 15.6 ng/mL for treated versus a mean of 22.3 for untreated HIV patients	[12, 22, 29–37]
Hepatitis C virus	Pegylated interferon combined with ribavirin	Blood	The rate of response was twofold higher among patients with pretreatment neopterin levels <16 nmol/L than in patients with levels ≥16 nmol/L	[38]
*Mycobacterium tuberculosis*	Antituberculotic treatment	Blood, urine	Levels declined on twice weekly measurements in all monitored patients with pulmonary tuberculosis on treatment and fell to below tolerance limits within 10 weeks of treatment in 6/10 patients	[39–41]
*Plasmodium falciparum*	Chloroquine	Urine	When clinical disease resolved within 3–7 days of treatment, neopterin levels normalized rapidly	[42]
*Schistosoma mansoni*	Praziquantel	Blood	Serum levels normalized on treatment	[43, 44]
